# Effects of Acute Caffeine Ingestion on Morning Strength, Power and Repeated Sprint Measures in Males: A Standardized Approach

**DOI:** 10.3390/nu18152541

**Published:** 2026-08-04

**Authors:** Sophie J. Vickery, Magali Giacomoni, João P. S. Agulhari, Theo Batten, Olivia G. Crozier, Dylan Goorwappa, Benoit Mauvieux, Samuel A. Pullinger, Neil Chester, Stuart J. Hesketh, Ben J. Edwards

**Affiliations:** 1 Research Institute for Sport and Exercise Sciences (RISES), John Moores University (Liverpool), Byrom Street, Liverpool L3 3AF, Merseyside, UK; s.vickery@2022.ljmu.ac.uk (S.J.V.); j.agulhari@2022.ljmu.ac.uk (J.P.S.A.); t.batten@2022.ljmu.ac.uk (T.B.); samuel.pullinger@inspireinstituteofsport.com (S.A.P.); n.chester@ljmu.ac.uk (N.C.); 2 Laboratory Youth Physical Activity Sport and Health (J-AP2S), University of Toulon, 83130 La Garde, France; magali.giacomoni@univ-tln.fr; 3 UR 7480 VERTEX, UFR STAPS, Université de Caen Normandie (UNICAEN), Normandie Université, 14000 Caen, France; benoit.mauvieux@unicaen.fr; 4 Sport Science Department, Inspire Institute of Sport, Vidyanagar, Bellary 583275, India; 5 School of Medicine and Dentistry, University of Lancashire, 135A Adelphi Street, Preston PR1 7BH, Lancashire, UK; shesketh5@lancashire.ac.uk

**Keywords:** placebo effect, RSA, RPE, diurnal variation, core temperature

## Abstract

Background/Objectives: Repeated sprint performance is typically reduced during the morning because of circadian-related changes in physiological function and muscle strength. Acute caffeine supplementation may attenuate these decrements; however, evidence remains inconsistent. This is partly because of inadequate control of chronobiological and methodological confounders. This study investigated the effects of acute morning caffeine ingestion on repeated sprint performance, physiological responses, strength, power and mood under rigorously standardized chronobiological conditions. Methods: Seventeen recreationally active, low habitual caffeine-consumer males completed six laboratory visits comprising three familiarization sessions and three experimental trials in a performance grouped, randomized for starting condition, counterbalanced, double-blind, placebo-controlled, crossover design. Participants ingested either caffeine (CAFF; 300 mg, 2.5–5.3 mg·kg^−1^), placebo (PLAC) or no-pill control (NoPill) 60 min before testing (07:30 h). Experimental sessions included assessments of mood, caffeine withdrawal symptoms, intra-aural temperature, grip strength, squat jump, countermovement jump and a 10 × 20 m repeated sprint protocol. Heart rate, ratings of perceived exertion (RPE), thermal comfort, blood lactate and blood glucose were assessed throughout the protocol. Data were analyzed using repeated-measures general linear models. Results: Caffeine did not significantly improve repeated sprint performance compared with PLAC or NoPill, although a trend towards faster 20 m sprint times was observed (3.39 ± 0.19 s vs. 3.43 ± 0.18 s and 3.41 ± 0.19 s, respectively; *p* = 0.070). Caffeine significantly reduced RPE (*p* = 0.029) but did not alter heart rate, blood lactate, blood glucose, thermal comfort, intra-aural temperature, mood, sleepiness, caffeine withdrawal symptoms nor lower-body power outcomes. Conclusions: Acute morning ingestion of 300 mg caffeine reduced perceived exertion but did not produce a clear ergogenic effect on repeated sprint performance under rigorously standardized chronobiological conditions. These findings suggest that, in low habitual caffeine users, the central perceptual effects of caffeine are insufficient to overcome the physiological constraints limiting morning repeated sprint performance and highlight the importance of rigorous chronobiological standardization when evaluating nutritional ergogenic aids.

## 1. Introduction

Repeated sprint ability (RSA) is a fundamental determinant of performance in many intermittent sports, including football, rugby, hockey and basketball. Where decisive moments frequently require athletes to perform repeated maximal efforts interspersed with brief and incomplete recovery periods [1,2]. The ability to maintain sprint performance across successive bouts is therefore critical to successful match play and is influenced by the integrated contribution of neuromuscular function, metabolic capacity and central nervous system regulation [2,3]. Importantly, RSA exhibits a pronounced time-of-day dependence, with performance consistently lower during the morning than the late afternoon or evening [4,5]. Despite this, athletes are frequently required to train and compete during morning hours because of tournament scheduling, travel demands and environmental constraints [6,7]. Consequently, identifying practical strategies capable of attenuating these morning decrements in repeated sprint performance has become an important objective for athletes, coaches and practitioners, yet evidence supporting effective interventions remains limited [4,8].

The physiological basis of diurnal variation in repeated sprint performance is thought to reflect the circadian regulation of multiple interacting systems that collectively influence exercise capacity. Peak physical performance typically occurs during the late afternoon or early evening [8], coinciding with the acrophase (peak) of core body temperature [9,10]; whereas, performance is generally attenuated during the morning biological phase. These time-of-day differences are accompanied by alterations in neuromuscular activation, muscle contractile properties, muscle temperature, metabolic responses and central nervous system function, which collectively influence force production, power output and fatigue resistance during repeated high-intensity exercise [11,12,13,14]. Although the precise contribution of each mechanism remains uncertain, converging evidence suggests that the interaction between central and peripheral physiological processes underpins the reduced repeated sprint performance observed during the morning [4,8]. Consequently, interventions capable of attenuating these circadian-related physiological limitations may provide meaningful benefits for athletic performance during morning training and competition.

Among the nutritional strategies proposed to mitigate morning reductions in exercise performance, acute caffeine ingestion is one of the most widely used and extensively investigated ergogenic aids [15,16]. Caffeine (1,3,7-trimethylxanthine) primarily exerts its ergogenic effects through antagonistic activity on adenosine receptors within the central nervous system, increasing arousal and neurotransmitter activity and reducing perception of fatigue. Leading to increased arousal, reducing perceptions of fatigue and enhancing neurotransmitter activity including dopamine and noradrenaline, which collectively promote wakefulness, attention and motor drive [17,18,19]. These mechanisms provide a strong physiological rationale for caffeine as a potential countermeasure to the circadian-related reductions in physical performance observed during the morning. Indeed, caffeine has consistently been shown to improve neuromuscular [14], time-trial performance [20] and exert a small beneficial effect on muscular endurance performance [21]. However, evidence relating to repeated sprint performance appears to depend on the caffeine naivety of the population, measure (set distance or set time), exercise mode and the administered caffeine dose; a more pronounced enhancement was observed with doses ≥ 6 mg·kg^−1^ body weight within cycling-based repeated sprint performance compared with running-based protocols [15,22,23,24,25,26,27]. Consequently, caffeine seems to represent a biologically plausible intervention capable of offsetting the physiological constraints associated with morning exercise performance.

Despite this strong physiological rationale, evidence regarding the effects of caffeine on repeated sprint performance, particularly during the morning, remains limited to only two experimental studies in adult males [28,29]. Previous investigations have differed substantially in caffeine dose and timing, habitual caffeine consumption, familiarization procedures, placebo design and statistical power, all of which represent recognized sources of bias when evaluating ergogenic interventions [15,30,31]. Furthermore, many studies have inadequately controlled chronobiological variables known to influence exercise performance, including chronotype, sleep quality, prior light exposure, habitual training time, season and environmental conditions [32,33]. Recent systematic reviews have similarly concluded that insufficient methodological standardization remains a major limitation within the chronobiology literature, contributing to bias in outcome measurement and reducing confidence in reported time-of-day effects [8,34,35]. Consequently, there remains a need for rigorously controlled investigations that minimize confounding factors when evaluating whether caffeine can meaningfully attenuate morning decrements in repeated sprint performance.

Therefore, the primary aim of this study was to determine whether acute morning caffeine ingestion could attenuate circadian-related reductions in repeated sprint performance under rigorously standardized chronobiological conditions. Secondary outcomes included measures of muscular strength and power, mood, perceived exertion and physiological responses to provide mechanistic insight into any observed changes in repeated sprint performance. We hypothesized that acute caffeine ingestion (CAFF) in a caffeine naive cohort would improve morning repeated sprint performance and reduce perceived exertion compared with both placebo (PLAC) and no-pill (NoPill) control conditions.

## 2. Materials and Methods

### 2.1. Participants

Seventeen male adults from biological sex and gender who were classified as “recreationally active” by the “Participant classification framework” [36] participated in the present study. We originally recruited 18 but one dropped out due to injury not associated with the research project. Participants completed a 7-day habitual food/fluid diary and weighed food intake and a 7-day habitual sleep recording using actigraphy (Motionwatch 8, CamnTech, Co., Dublin, Ireland), in addition to a sleep diary as a secondary measure (Table 1). The food diary that was analyzed with Nutritics^®^ analysis software Version 6.23 this process was conducted by a SENr registered Sports and Exercise Nutritionist and key information such as daily calories, macro and micronutrient intake as well as levels of caffeine and water intake (see Table 1 [37]). This information was compared against recommended daily intake amounts, and this population consumed more protein, Vitamin B_6_ and water, but less carbohydrates than recommended (Table 1). Key sleep variables presented were Actual sleep-time (h:min), Time-in-bed (h:min), Sleep-latency (h:min) and Sleep-Efficiency (%). We originally opened participation to females, but no females volunteered. All participants were recruited from the University Sport Science department student population. This study was registered retrospectively on ClinicalTrials.gov (registration number: NCT07469852), approved on 9th March 2026. At the time of study initiation, the research team did not realize that prospective registration was required for a non-CTIMP, nutrition-based crossover study involving healthy participants and sporting performance outcomes. Upon becoming aware of journal and transparency requirements, the study was registered to ensure public accessibility and accountability. The study protocol, eligibility criteria, intervention conditions, primary and secondary outcome measures were defined prior to participant recruitment and were not modified following study commencement. No changes were made to the study design or outcomes after registration.

Inclusion criteria were healthy males (18–30 years), injury-free, with previous team-based sport participation experience such as Football, Rugby or Hockey (≥5 years) and were used to rapid acceleration in speed and recovery hence repeated sprinting. Recruiting participants with this specific type of exercise history meant that the main measurement test (repeated sprinting) was not novel and the known neuromuscular facilitative responses, which are typically associated with acute increases in muscular strength and power amongst untrained individuals due to neural adaptations and responses were reduced [6]. Participants habitually retired between 22:00 and 23:30 h and rose at 06:00–07:30 h and agreed to retire to bed at 22:30 and rise at 06:30 h (like their normal habits and close to their mean habitual opportunity to sleep time of ~08:19 h:min). None of the participants were receiving any pharmacological treatment (including non-steroidal anti-inflammatory drugs, NSAIDs) throughout the study period. Habitual caffeine consumption was assessed using the caffeine consumption questionnaire (CCQ) and those with >150 mg per day were excluded [38] (mean values were 122.9 mg, see Table 1). Further, all participants expressed no preference to training regarding time-of-day by a weekly self-reported 2-week training diary. Exclusion criteria included depressed mood (from the Beck depression inventory [39]), poor sleep quality (a Pittsburgh sleep quality index global score >5 [40]), recent shiftwork or travel across multiple time-zones and ‘extreme’ chronotype (assessed via the Composite Morningness Questionnaire [41]) and risk factors and/or symptoms of cardiovascular disease. Through interviews, it was established that the participants had minimal knowledge of the effects of time-of-day or time-since-sleep on human performance. Verbal explanation of the experimental procedure was provided to everyone; this included the aims of the study, the possible risks associated with participation, and the experimental procedures to be utilized. Any questions were answered. Individuals then provided written, informed consent before participating in the study. The experimental procedures were approved by the Human Ethics Committee at Liverpool John Moores University (registration number: M24_SPS_4442). The study was conducted in accordance with the ethical standards of the journal and complied with the principles of the Declaration of Helsinki.

### 2.2. Experimental Design

**Figure 1:**
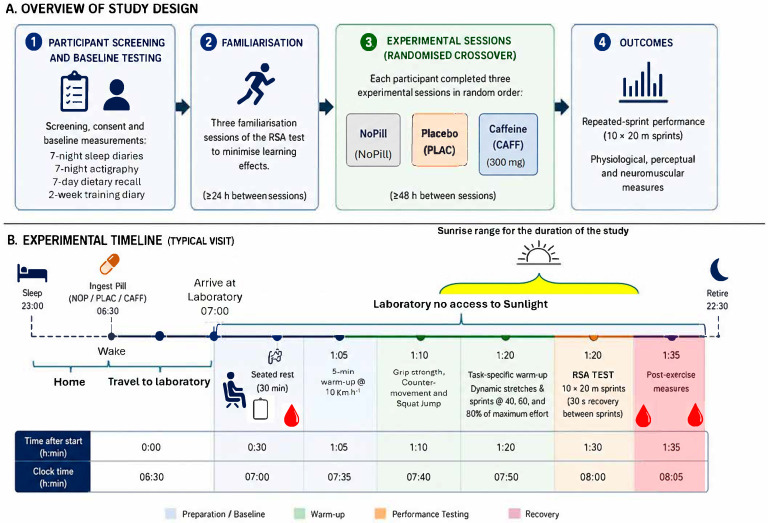
Schematic representation of the research design and experimental protocol undertaken by participants in the investigation. Pills were taken upon wakening; first timeline starts from arrival at the laboratory (Lab). The thermometer represents T_IA_ measurements, the clipboard where subjective resting ratings were asked, and red drops represent where blood was taken. Sunrise for the duration of the study was from 07:41 to 08:03 h considering refraction.

The participants visited the physiological laboratory at JMU (Liverpool, UK) on six occasions, with environmental conditions of wet bulb globe temperature 13–17 °C, dry temperatures of 19–21 °C, 26–44% humidity, barometric pressure of 990–1022 mmHg and ambient light of 750 lux. Each participant completed (i) three familiarization sessions. The remaining sessions consisted of (iii) three experimental sessions, all at least 72 h apart as a standardized recovery period (Figure 1). All the trials were performed with only one participant at a time. For the 24 h period before the experiment, participants were asked to replicate the diet, feeding pattern and macro/micronutrient content but in the absence of caffeine intake from the habitual 7-day dietary recall, based on RDA values for a balanced diet [37]. The experimental trails included (a) NoPill control (NoPill), (b) 300 mg caffeine ingestion (CAFF) and (c) placebo (maltodextrin, PLAC) at 07:00 h, participants retired the previous night around 22:30 h, woke up at 06:30 h and arrived at the laboratory at 07:00 h in a fasted state. Participants were instructed to avoid training or intense physical activity 48 h prior to any of the visits, otherwise, participants maintained their normal daily routines. In total the three caffeine capsules contained 300 mg of caffeine anhydrous and the remaining space was occupied by maltodextrin (Applied Nutrition LTD, Knowsley, UK) and placebo capsules were made in the department and contained maltodextrin (~1.55 g, Sport supplements Ltd. t/a BulkTM, Colchester, UK). Researchers and participants were blinded to the supplement schedule and pills were provided in a plastic bottle with instructions to consume with water 30 min before retiring. Both CAFF and PLAC were lightly dusted with maltodextrin to create a similar taste, had similar weight (0.8 g/capsule) and were 00 size. At the end of the experiment the order of treatment was revealed to the researchers by an author (BE).

All the trials were performed with only one participant at a time, but with a staggered start so one participant came in at 06:30, the next 06:45 h and the last for that morning’s session at 07:00 h. This scheduling for participants was kept for all sessions. Participants were equally allocated, based on their 20 m repeated sprint mean finishing times into three groups (A, B and C) by listing participants’ times from the third familiarization in order from the fastest to the slowest and assigning letters A, B and C in order. This then created 6 rows with 18 participants in, we then randomly allocated individuals from each row into condition 1, 2 or 3 based on the lead author [BJE] picking three numbers from a box and repeating the process 5 more times. Having assigned the individual to a starting condition the incidence of the learning effect was minimized by assigning the experimental conditions in counterbalanced order [33]. The present study was carried out between the months of 17th November to 20th February (UK Autumn to Winter), with morning sunlight exposure limited to <80 Lux before arriving at the laboratory. To measure this the lead author [BE] took readings when the participants entered the campus from the roof of the building, which was deemed as not substantially affected by street lighting or light pollution (where the Astronomy department has its weather station), using a factory-calibrated digital lux metre (Lux meter LX-1330B with 0 to 200,000 lux output range, Drmeter, Chicago, IL, USA). The sunrise range for the duration of the study was 07:41 to 08:03 h considering refraction (Figure 1).

### 2.3. Protocol and Measurements: Familiarization Session

The participants completed three familiarization sessions of all strength/power, repeated sprint and questionnaire measures before being considered ready to participate in the study. A coefficient of variation (CV) value less than or equal to 10% for variables between the third and second measures was used as criteria for familiarization in alignment with the recommendations given [42]. Delta change, random variation (CV) and systematic bias (paired *t*-test) for the repeated sprint variables (20 m mean time, 5 m mean time and fatigue index were (−0.02 s, 2.75%, *p* = 0.392; −0.01 s, 6.77%, *p* = 0.666; 0.89%, 3.66%, *p* = 0.329). Familiarization sessions took place at 12:00 h over a three-week period and finished one week before the study commenced to minimize learning effects. Participants arrived 0.5 h before the start of the test and rested in a seated position, to minimize the influence of prior muscle activity. On the first visit basic measures for height, mass and body fat were assessed using a stadiometer (Seca model 213 standard portable stadiometer), scales (Seca model 875 standard portable scale, Birmingham, UK) and through ISAK skinfold assessment estimating subcutaneous adipose tissue and overall body adiposity ([43] Table 1). During the familiarization and experimental sessions participants completed the different tests in the following order after a 5 min warm-up at 10 km·h^−1^ on a treadmill (Pulsar, H/P cosmos, Munchen, Germany): (i) isometric hand grip test, (ii) squat jump, (iii) countermovement jump measures and (iv) repeated sprint test (Figure 1).

(i) Isometric grip test

Grip strength was assessed using a handgrip dynamometer (Takei Scientific instruments Company Ltd., Tokyo, Japan). Participants performed three maximal grip strength attempts with each hand in an alternating sequence (right hand, left hand) with the highest value recorded for each hand retained for analysis. For each attempt, participants began with the testing arm fully extended above the head alongside the ear while holding the dynamometer. Participants were then instructed to squeeze maximally in a controlled manner whilst lowering their arm until it was fully extended by their side.

(ii) Squat jump and (iii) countermovement jump measures

Squat jump (SJ) and countermovement jump (CMJ) performance were assessed using the OptoJump system (Microgate, Bolzano, Italia), with jump height recorded, using OptoJump software version 1.13 and subsequently analyzed. For SJ, participants began in an upright standing position with their hands placed on their hips to minimize upper body contribution. Participants performed a squat movement and immediately executed a maximal vertical jump without arm involvement. For CMJ, participants began in an upright standing position with their arms extended overhead. Participants then performed a rapid downward movement whilst simultaneously swinging their arms backwards before explosively extending upwards and swinging the arms overhead during takeoff.

(iv) Repeated Sprint Ability test (RSA)

The participants completed 10 × 20 m sprints on a biosynthetic runway using infrared timing gates (Brower TCi Timing system, Draper, UT, USA) with 30 s of active recovery after each sprint. Measures of 5 m and 20 m sprint times for the 10 sprints were recorded and later analyzed. Further indices were also measured: the best sprint time (RSA_BEST_), the mean sprint time (RSA_MEAN_), the total time (RSA_TT_), the percentage sprint decrement [RSA_DEC_ = ((RSA_BEST_ − RSA_MEAN_ × 10)/(RSA_TT_ × 10) × 100)] and the percentage difference from the best and worst sprint during the RSA test [RSA_CHANGE_ = ((Worse − Best time)/(Best time) × 100) [44]]. As well as fatigue index measured using two methods (1) [((Best time/Worse) × 100))−100] [4] and by (2) [(sprint 1 + 2 times) − (Sprint 9 + 10 times)/((sprint 1 + 2 times)] × 100] [28].

### 2.4. Experimental Protocol and Measurements

The experimental sessions took place a week after the last familiarization, with 72 h recovery between the three experimental conditions (i.e., NoPill, CAFF and PLAC). Participants arrived at the laboratory 5 min before the start of the test, participants then rested for 30 min in supine position to assess resting intra-aural temperature (T_IA_, Genius 1000, Mark 2, Sherwood, Nottingham, UK). For the last 5 min of the resting period T_IA_ values were taken twice and the average value recorded [45]. Participants were fitted with HR chest strap monitors (Polar H10, Polar, IOS 17, Kempele, Finland) with data collected on the phone Bluetooth 4.0 Polar app (Polar Flow app, Polar, Kempele, Finland) while rested. And completed profile of mood states questionnaire (POMS [46]), their subjective rating of sleep (using sleep questions from the Liverpool Jet lag Questionnaire [47]), effort (0–10, 0 = no effort and 10 = maximal effort) and rate of perceived effort (RPE [48]). Lastly, caffeine withdrawal symptoms were assessed using a caffeine withdrawal questionnaire [49] as well as hunger and satiety from a 0 (Not at all) to 10 (Extremely) scale [50]. Fingertip capillary blood samples were collected at rest, immediately post and 5 min after repeated sprints. And analyzed for lactate and glucose concentrations levels by table-top analyzer (Biosen C-Line Glucose and Lactate Analyzers, EKF Diagnostics, Cardiff, UK). Fifteen minutes into this pre-exercise resting period, participants completed the battery of sleep and mood tests. Participants then warmed up on a treadmill for 5 min at 10 km·h^−1^, completed a series of dynamic stretches and undertook practice 20 m sprints at 40, 60 and 80% of maximum effort. Thereafter, participants were directed to complete the 10 × 20 m sprints with 30 s of recovery as fast as possible, with continuous verbal motivation throughout. Values for HR, RPE, effort, 5 m and 20 m times were collected every sprint. Completion times 20 m were not given to participants, to stop competition with their previous performance [33,51].

### 2.5. Statistical Analysis

The main research variable in the present study was repeated sprint performance measures (total 20 m time and mean 20 m time). The coefficient of variance (CV) in similar protocols with test–retest has been reported to be reliable <2%, like our familiarization trials 2.7% [52]. Eleven participants were estimated to provide 80% statistical power (*p* < 0.05), with effect size (ES) of 0.95 [28,29] for paired *t*-test with 2 tails. The 90% figure of the statistical power required 14 participants and therefore we recruited 18 participants to allow for a dropout. The sample size estimation was performed using G*Power 3.1 [53]. Data were analyzed using the Statistical Package for Social Sciences version 31 (SPSS, Chicago, IL, USA). All data were checked for normality using the Shapiro–Wilk test. General linear models were used to analyze all measurements collected; the significance of interactions and main effects were evaluated by ANOVA. Sphericity violations were investigated by Mauchly’s test for sphericity and ε corrected by Greenhouse–Geisser (ε < 0.75) or Huynh–Feldt (ε > 0.75) and main effects by Bonferroni pairwise comparisons. All data is presented as means ± standard deviation (SD), unless stated otherwise. Significance was set at *p* ≤ 0.05. *p* values between 0.05 and 0.10 were considered to indicate a statistical trend [54]. Effect sizes are referred to as partial eta squared values (η^2^p), with values of 0.01, 0.06 and 0.14 corresponding to a small, medium and large effect, respectively—thresholds proposed by Cohen [55]. Ninety-five percent confidence intervals (95% CI) and mean differences between pairwise comparisons are reported where suitable. Mean differences or change between pairwise comparisons are given the symbol in the text.

## 3. Results

### 3.1. Rest and Post Warm-Up Variables

There were no significant main effects of experimental condition for T_IA_ values (*p* = 0.709, η^2^p = 0.02 Table 2). There was a main effect for “rest to post warm-up”, where T_IA_ values increased after the treadmill warm-up (0.18 °C CI = 0.08 to 0.28 °C, *p* = 0.002; large effect size, η^2^p = 0.46). There were no significant interactions such that the profiles for condition from pre to post were the same (*p* = 0.433, Table 2).

### 3.2. Grip Strength, Squat Jump and Countermovement Jumps

There was a main effect for condition for left grip strength, where grip strength was higher in PLAC than in NoPill (2.3 N, *p* = 0.050, Table 2), but there was no difference compared to the CAFF condition. No other measures for grip strength, squat jump or countermovement jump showed a main effect for conditions.

### 3.3. Response to Repeated Sprint

**Figure 2:**
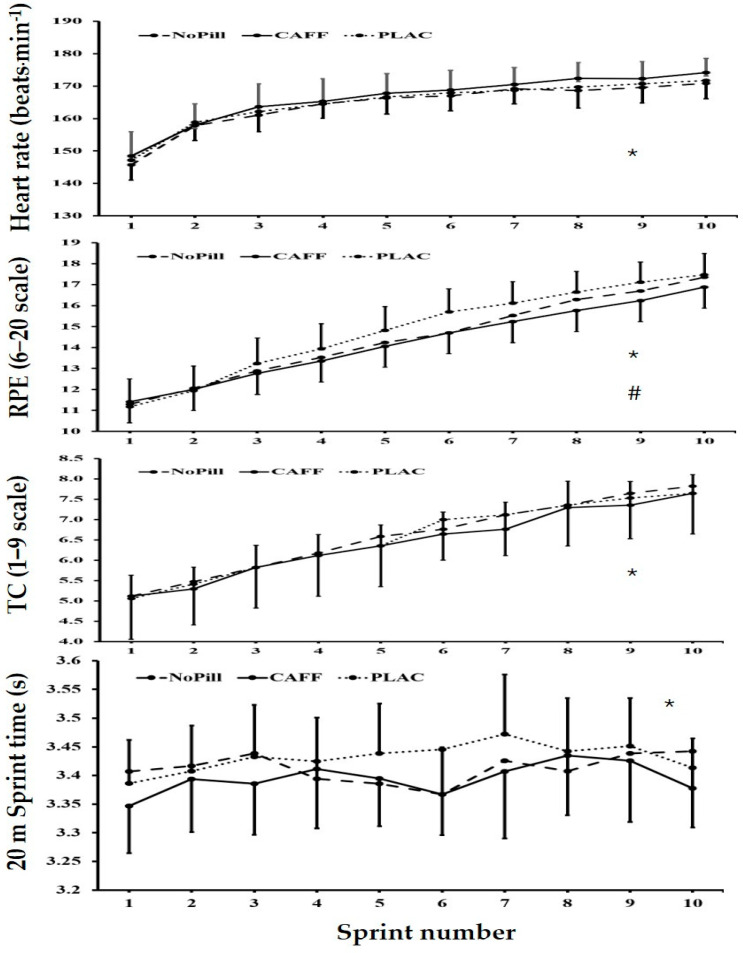
Individual and CI values of 20 m split times, heart rate, thermal comfort (TC) and rating of perceived exertion (RPE) for the three experimental conditions (NoPill, CAFF or PLAC). # denotes main effect for condition, * denotes main effect for sprint as shown by Bonferroni pairwise comparisons (*p* < 0.05).

There was a significant main effect for rating of perceived exertion (*p* = 0.029, large effect size, η^2^p = 0.20, Table 2) where pair-wise analysis showed lower values in the CAFF than the PLAC or NoPill conditions (0.58 AU [arbitrary units], *p* < 0.001 and 0.22 AU, *p* = 0.017, respectively (Table 2). There was a trend for 20 m times to be faster in CAFF vs. NoPill and PLAC conditions (*p* = 0.070, large effect size = 0.18), with a trend for RSA_CHANGE_ showing greater values in the CAFF condition (*p* = 0.067, large effect size, η^2^p = 0.16). A significant main effect for number of sprints was found for heart rate, RPE, thermal comfort, lactate and blood glucose (*p* ≤ 0.017), where heart rate, RPE and thermal comfort increased from the rest to the end of the RSA exercise (Figure 2). And blood glucose and lactate levels increased from rest to exercise. There was no significant interaction between condition and sprint.

### 3.4. Hunger Scales

There was a significant main effect of experimental condition for fullness (*p* = 0.004, η^2^p = 0.31; Table 2), with greater fullness reported under caffeine compared with NoPill and PLAC (1.4 AU, 95% = 0.31 to 2.40 AU, *p* = 0.014; 1.7 AU, 95% = 0.6 to 2.8 AU, *p* = 0.004). There were no significant main effects of experimental condition for hunger, desire for savoury food, desire for sweet food, physical tiredness, sleepiness/drowsiness, energy/alertness (*p* > 0.05). There was a trend for lethargy/sluggishness values to be lower in the CAFF condition than NoPill or PLAC.

### 3.5. Resting Profile of Mood States, Caffeine Withdrawal and Sleep Questionnaires

There was no significant effect of condition for any mood profiles (vigour, anger, tension, calm, happiness, confusion, depression or fatigue), ratings of alertness or fatigue, caffeine withdrawal symptoms, Stanford sleepiness questionnaire nor for sleep questionnaire variables (see Table 3). There was a trend for alertness upon arriving at the laboratory being higher in CAFF than NoPill or PLAC (*p* = 0.093, η^2^p = 0.14).

## 4. Discussion

The present study investigated whether acute morning ingestion of a moderate dose of caffeine (300 mg) could attenuate the well-established reduction in repeated sprint performance under rigorously standardized chronobiological conditions. The principal finding was that caffeine ingestion (CAFF) did not elicit a clear ergogenic effect on morning repeated sprint performance compared with either placebo (PLAC) or no-pill (NoPill) control conditions. Although a trend towards faster 20 m sprint times was observed to be faster in CAFF vs. NoPill and PLAC conditions (3.39 ± 0.19 s vs. 3.43 ± 0.18 s and 3.41 ± 0.19 s; *p* = 0.070, η^2^p = 0.18), representing an approximately 1.2% faster sprint time than PLAC (Table 2). However, from the familiarization data considering the last session compared to the penultimate one for 20 m mean time, the change in values were 0.02 s or 0.6%. Hence the sensitivity of the test for this outcome variable was half that of the observed effect, the main implication is that the test may not have been capable of reliably detecting effects of the magnitude that was observed and any effect of caffeine is likely to be modest. In a recent systematic review, diurnal variations on performance indices in repeated sprint tests were 3.5–5.8% for distance covered, average and peak velocity and average power for non-motorized treadmill tests [4].

In cycling, power output differed across all sprints by 6.0% in one study and 8.0% for the first sprint only in five studies [4]. All four studies measuring power decrement values (i.e., rate of fatigue) established differences up to −4.0% and two out of five studies established total work to be significantly higher by −8.0% in the evening than morning [4]. To quantify the ability to resist fatigue during repeated sprints, researchers have tended to use one of two terms, the fatigue index (FI) or the percentage decrement score (%). In the current study, we found a trend of RSA_CHANGE_ to be greater by −1.1 to −1.3% in the CAFF vs. NoPill or PLAC conditions (*p* = 0.067, large effect size η^2^p = 0.16). Like the outcome variable mean 20 m time, the sensitivity of this test was 0.5%. The two methods of fatigue index commonly used in the literature, however, showed no effect (Table 2 [4,56]), reflecting the poor sensitivity of such measures using running tracks rather than non-motorized treadmills where time rather than distance is prescribed. In contrast, CAFF significantly reduced ratings of perceived exertion compared with both PLAC and NoPill conditions (*p* = 0.029, η^2^p = 0.20), despite no corresponding changes in heart rate, blood lactate, blood glucose or thermal comfort throughout the repeated sprint protocol (Figure 2; Table 2). Such a trend could be due to a slight but not significant increase in 20 m sprint time in CAFF as a similar trend was also observed for this variable with large effect size (3.39 ± 0.19 s vs. 3.43 ± 0.18 s and 3.41 ± 0.19 s for PLAC and NoPill, respectively; *p* = 0.070, η^2^p = 0.18). Caffeine ingestion also exerted minimal influence on secondary outcomes, with no meaningful effects on lower-body power, mood state, sleepiness, or caffeine withdrawal symptoms measures. Similarly, left grip strength values were higher in PLAC than NoPill (2.3 N, *p* = 0.050, Table 2) but showed no difference with the CAFF condition. The participants, when asked, did not know if they were on the caffeine or placebo condition, but due to being students in Sport Sciences and nutrition in sport degree programmes they knew of the effects of caffeine as an ergogenic. Improvement could therefore have been due to expectancy effect mechanisms [15]. Collectively, these findings suggest that, in low habitual caffeine users tested during the early morning, acute ingestion of a moderate dose of caffeine favourably influenced perceptual responses to exercise but was insufficient to overcome the physiological constraints limiting repeated sprint performance. The lack of an improvement in repeated sprints with caffeine rejects the first hypothesis that caffeine aids short-term performance. The lower RPE in the CAFF condition compared to NoPill or PLAC without an increase in performance confirms the second hypothesis that caffeine would reduce perception of effort which could aid longer exercise. The absence of a clear ergogenic effect of acute caffeine ingestion on repeated sprint performance suggests that adenosine receptor antagonism alone may be insufficient to overcome the complex physiological constraints associated with morning exercise performance [15,17]. The major factors influencing physiological performance in the morning, include sleep inertia, circadian rhythms and homeostatic drive [6]. As well as time on task and the level of difficulty or cognitive domain [16,34]. In our population of intermediate types who were caffeine naive, the expectation was that the morning would produce the lowest performance on the circadian rhythm. At this time homeostatic pressure (drive to sleep) would be weak as participants had only been awake for an hour at the time of physiological testing, so levels on adenosine release in the brain by activity should be low and sleep inertia should not be present. Further, similar states of alertness, tiredness and sleepiness were reported. Therefore, reducing the effectiveness of caffeine’s main ergogenic mechanism on performance. We found self-reporting of stomach fullness higher in the caffeine than the NoPill or Placebo condition (4.8 ± 2.2 vs. 2.7 ± 1.6 and 2.9 ± 2.0 AU respectively). The scale for this question is from a 0–10 cm with “O” being “Not at all” and 10 cm being “extremely”. Given that the amount of literature investigating caffeine naive populations is small and a recent review found insufficient evidence to determine coffee or caffeine’s role in inducing appetite perceptions, interpretation of this finding although intriguing should be treated cautiously [50]. Similarly, as the participants were fasted the effect of this perception on the performance, RPE and other outcomes is unknown but warrant further investigation.

Repeated sprint ability is dependent upon the integrated contribution of neuromuscular function, anaerobic metabolism, central motor drive and fatigue resistance [2,3,4]. Several of which are influenced by circadian rhythms and exhibit suboptimal function during the morning biological phase [4,57]. Recent evidence from well-controlled chronobiology studies has demonstrated that these morning reductions persist even after rigorous standardization of sleep, chronotype and nutritional status [4,16,33], as well as habitual training time and environmental conditions [33,58,59]. In the present study, acute ingestion of 300 mg caffeine produced only a modest trend towards faster sprint performance (~1.2% versus PLAC, large effect size of 0.18), without corresponding changes in heart rate, blood lactate, blood glucose or thermal comfort. Collectively, these findings suggest that although caffeine may modestly enhance central nervous system activation, its effects were insufficient to meaningfully alter the peripheral physiological determinants limiting repeated sprint performance during the morning. Rather than questioning the ergogenic properties of caffeine per se, these findings indicate that the biological constraints imposed by circadian physiology may not be readily overcome by a single acute nutritional intervention.

The present findings are consistent with the increasingly nuanced literature describing the ergogenic effects of caffeine during repeated sprint exercise [15,23]. Although the International Society of Sports Nutrition position stand continues to conclude that caffeine is an effective ergogenic aid for endurance exercise, prolonged intermittent activity and muscular endurance, typically at doses of 3–6 mg·kg^−1^ body mass [15], evidence relating specifically to repeated sprint performance remains considerably less consistent. Recent systematic reviews and meta-analyses report small overall improvements in repeated sprint ability following acute caffeine ingestion but also highlight substantial heterogeneity arising from differences in participant characteristics, habitual caffeine intake and supplementation protocols, as well as sleep habits, sprint testing procedures and outcome measures being tested [23,27]. Similarly, Zhang et al. [30] concluded that the ergogenic response to caffeine may itself be influenced by time-of-day but emphasized that current evidence remains limited by inconsistencies in testing windows, chronotype control, sleep standardization and exercise modality. Consequently, while caffeine clearly possesses ergogenic potential [59], the magnitude of its effect during repeated sprint exercise appears highly context-dependent and influenced by both experimental design and biological timing.

Importantly, relatively few studies have examined the ergogenic effects of caffeine using a rigorously standardized chronobiological protocol [33]. Agulhari et al. [16], employing a highly controlled experimental design comparable to the present investigation, where time on task was 3 s, reported improvements of ~9% in morning muscle isometric maximum voluntary contraction peak-force values following acute caffeine ingestion as well as observing selected benefits in cognitive function. Together with our recent observations demonstrating persistent morning reductions in neuromuscular and endurance performance despite comprehensive behavioural and environmental standardization [59], the present findings support the emerging concept that biological timing exerts a robust influence on exercise performance that dependent on the task, is not easily offset by acute caffeine supplementation alone. Rather than suggesting caffeine is ineffective, these collective findings indicate that the magnitude of its ergogenic effect during morning exercise may be smaller than that implied by the broader literature when chronobiological and behavioural confounders are rigorously controlled.

Despite the absence of a clear improvement in repeated sprint performance, caffeine significantly reduced ratings of perceived exertion (Table 2), suggesting that its central effects on exercise perception may be more robust than its influence on objective physical performance. This finding is consistent with the proposed mechanism of caffeine as an adenosine receptor antagonist, reducing central fatigue and increasing arousal; thereby lowering the conscious perception of effort required to perform a given task [15,17,19]. The dissociation observed between perceived effort and exercise performance is noteworthy and suggests that reductions in RPE do not necessarily translate into measurable improvements in repeated sprint ability, particularly when performance may be constrained by peripheral physiological factors associated with the morning biological phase [25,29]. Similar dissociations have been reported previously, whereby caffeine consistently lowers perceived exertion despite variable effects on objective performance outcomes, particularly during short-duration, high-intensity exercise [15,60,61,62]. Therefore, while caffeine may improve the subjective experience of exercise, these perceptual benefits alone appear insufficient to overcome the integrated neuromuscular and metabolic limitations that underpin repeated sprint performance during the morning. Nevertheless, a reduction in perceived exertion may still have practical value during training or competition, potentially allowing athletes to tolerate demanding exercise with less subjective fatigue, even when measurable improvements in performance are small or absent.

An important consideration when interpreting the present findings is the rigorous chronobiological standardization employed throughout the experimental protocol. Growing evidence indicates that methodological factors including chronotype, habitual training time, sleep quality, nutritional status and environmental conditions, as well as familiarization procedures exert meaningful influences on both exercise performance and the observed response to ergogenic interventions [33]. Consequently, inadequate control of these variables may either exaggerate or obscure the true physiological effects of caffeine, contributing to the inconsistent findings reported across the literature. The present study adopted the standardized methodological framework proposed by Edwards et al. [33], incorporating comprehensive behavioural and environmental control alongside placebo and no-pill comparator conditions to minimize potential sources of bias. The framework allows critical comparison between similar research, to this end we have compared our and the two similar studies against this framework (Table 4). Importantly, recent investigations employing similarly rigorous protocols have consistently reported more modest ergogenic responses than many earlier studies [14,16], suggesting that careful chronobiological standardization may provide a more accurate estimate of caffeine’s true efficacy during morning exercise.

### 4.1. Limitations

Several considerations should be acknowledged when interpreting the present findings. First, although the use of a fixed caffeine dose (300 mg) reflects a practically relevant supplementation strategy, the relative dose administered varied according to individual body mass and may have contributed to inter-individual variability in the ergogenic response. Secondly, participants were intentionally recruited as low habitual caffeine consumers to minimize the confounding effects of caffeine tolerance and withdrawal, thereby strengthening internal validity. However, these findings may not be directly generalizable to athletes with higher habitual caffeine consumption. Furthermore, although we opened the recruitment to both males and females, only healthy young males participated, limiting extrapolation to female athletes or older populations, in whom circadian physiology and caffeine metabolism may differ. The present investigation also focused exclusively on morning exercise and therefore cannot determine whether similar responses would be observed later in the day when physiological capacity is generally enhanced. Further, although we sort to restrict daylight exposure by conduction the experiment in winter in the UK, we did not measure the exposure of light of the participants from waking, during travel to entering the laboratory. Finally, although perceptual and physiological outcomes provided valuable mechanistic insight, direct assessment of neuromuscular activation or central nervous system function, e.g., electromyography, transcranial magnetic stimulation or electroencephalography was beyond the scope of the present study and would further strengthen mechanistic interpretation.

### 4.2. Practical Implications and Future Research

From an applied perspective, the present findings suggest that athletes and practitioners should not assume that acute caffeine supplementation will reliably overcome the physiological constraints associated with morning repeated sprint exercise, even in low habitual caffeine users. Although caffeine reduced perceived exertion, this perceptual benefit did not translate into a meaningful enhancement of repeated sprint performance under rigorously controlled conditions, emphasizing that biological timing may fundamentally influence the magnitude of an ergogenic response. Consequently, nutritional strategies should not be considered in isolation but rather within the broader physiological context in which they are implemented.

Future research should therefore move beyond the simplistic question of whether caffeine is ergogenic and instead determine when, for whom, and under which biological conditions caffeine is most effective. This will require studies incorporating individual chronotype, habitual caffeine intake, sex, training status and biological timing alongside dose–response investigations using relative (mg·kg^−1^) supplementation strategies. Furthermore, combining caffeine with interventions specifically designed to modify circadian physiology, such as exercise timing, strategic light exposure, thermal interventions or structured warm-up protocols, may prove more effective than caffeine supplementation alone. The present findings support a shift away from a ‘one-size-fits-all’ approach to sports nutrition towards precision chrono-nutrition strategies that recognize biological timing as a fundamental determinant of ergogenic efficacy and athletic performance.

## 5. Conclusions

Acute ingestion of a moderate dose of caffeine (300 mg) did not produce a clear ergogenic effect on repeated sprint performance during morning exercise under rigorously standardized chronobiological conditions, despite reducing perceived exertion. This finding suggests that the central and perceptual effects of caffeine alone are insufficient to overcome the physiological constraints limiting morning repeated sprint performance. Further, in low habitual caffeine users, the central perceptual effects of caffeine may not be sufficient to overcome the physiological constraints by limiting repeated sprint performance during the morning biological phase. Importantly, the rigorous chronobiological standardization employed throughout the present study provides a robust estimate of the effects of acute morning caffeine ingestion under tightly controlled experimental conditions and highlights the importance of accounting for biological timing when evaluating nutritional ergogenic aids. In summary, this study contributes to the growing body of evidence demonstrating that the ergogenic efficacy of caffeine is context-dependent and should be interpreted within the framework of circadian physiology and rigorous methodological control.

## Figures and Tables

**Table 1:** Participants’ physical characteristics, baseline Actimitry and food intake of the (mean ± SD values).

	Variables	
**Physical characteristics**		
Age (yr)	20.6 ± 1.2	
Height (cm)	180.1 ± 8.1	
Mass (kg)	81.4 ± 15.5	
Body fat (%)	17.5 ± 3.5	
Chronotype	32.4 ± 5.3	
Languid/Vigorous	47.1 ± 6.1	
Flex/Rigidity	43.8 ± 6.5	
Athletic level (1–5)	2.2 ± 0.9	
Caffeine intake from questionnaire (mg)	122.9 ± 77.2	
**Baseline Actimitry**		
Retiring time (h:min)	22:54 ± 00:30	
Waking time (h:min)	07:36 ± 00:54	
Time-in-bed (h:min)	08:42 ± 00:54	
Sleep-latency (min:s)	22:56 ± 11:32	
Sleep-Efficiency (%)	85.2 ± 8.7	
**Baseline food intake**	**Participants intake**	RDA *
Daily calories (kcal)	2205 ± 470	2500 (*p* = 0.101)
Fats (g)	90 ± 83	97 (*p* = 0.485)
Protein (g)	**157 ± 40**	**56 (*p* < 0.001)**
Carbohydrates (g)	**202 ± 80**	**330 (*p* < 0.001)**
Zinc (mg)	8.4. ± 3.9	11 (*p* = 0.064)
Magnesium (mg)	232 ± 78	**400 (*p* < 0.001)**
Vitamin B6 (mg)	2.6 ± 1.8	**1.4 (*p* = 0.005)**
Water intake (ml)	**3879 ± 1150**	**2000** (*p* = 0.069)

* Denotes recommended daily values (RDA) from Nutrition Science Team [37]. **Bold** values denote significant values compared to RDA from a paired *t*-test.

**Table 2:** Mean ± SD values for all variables measured.

Variable	NoPill	CAFF	PLAC	Significance Condition (*p*, η^2^_p_, OP)	Significance of Pre-Post/Sprint Number (*p*, η^2^_p_, OP)	Interactions (*p*, η^2^_p_, OP)
**Temperature**						
Intra-aural temperature (°C)	36.66 ± 0.55	36.57 ± 0.41	36.58 ± 0.58	*p* = 0.709, 0.02, 8.7	*p* = 0.002, 0.463, 93.7	*p* = 0.433, 0.05, 14.4
**Strength and power**						
Right grip strength (N)	42.3 ± 6.9	42.8 ± 7.7	42.7 ± 7.6	*p* = 0.761, 0.02, 8.9		
Left grip strength (N)	37.8 ± 9.0	39.2 ± 8.8	40.1 ± 7.6 *	***p* = 0.046, 0.18, 59.9**		
Squat jump (cm)	34.1 ± 4.7	34.4 ± 5.2	33.8 ± 5.0	*p* = 0.422, 0.05, 18.9		
Counter movement jump (s)	38.8 ± 6.0	39.1 ± 6.6	38.7 ± 6.7	*p* = 0.794, 0.01, 7.8		
**Repeated sprint ability (10 × 20 m)**						
5 m time (s)	1.15 ± 0.09	1.16 ± 0.10	1.15 ± 0.08	*p* = 0.552, 0.04, 9.9	*p =* 0.485, 0.52, 24.6	*p* = 0.494, 0.07, 20.7
20 m time (s)	3.41 ± 0.19	3.39 ± 0.19	3.43 ± 0.18	*p* = 0.070, 0.18, 47.1	*p* = 0.294, 0.07, 25.2	*p* = 0.239, 0.08, 46.9
Fatigue index 1 (%)	−6.0 ± 2.4	−5.2 ± 2.1	−5.4 ± 2.2	*p* = 0.246, 0.08, 26.7		
Fatigue index 2 (%)	−0.83 ± 2.14	−0.89 ± 2.44	−1.05 ± 2.66	*p* = 0.912, 0.004, 5.9		
RSA_DEC_ (%)	−9.03 ± 0.01	−9.03 ± 0.01	−9.03 ± 0.01	*p* = 0.969, 0.001, 5.3		
RSA_CHANGE_ (%)	6.0 ± 2.6	7.1 ± 2.7	5.8 ± 2.5	*p* = 0.067, 0.16, 53.4		
Heart rate (beats·min^−1^)	164 ± 12	166 ± 13	165 ± 13	*p* = 0.602, 0.03, 12.7	***p* < 0.001, 0.90, 100.0**	*p* = 0.693, 0.04, 45.3
RPE (6–20)	14.5 ± 3.1	14.2 ± 3.0 *	14.8 ± 3.2	***p* = 0.029, 0.20, 67.2**	***p* < 0.001, 0.89, 100.0**	*p* = 0.063, 0.12, 68.4
Thermal comfort (1–9)	6.6 ± 1.3	6.4 ± 1.3	6.5 ± 1.3	*p* = 0.567, 0.04, 13.7	***p* < 0.001, 0.82, 100.0**	*p* = 0.613, 0.04, 23.3
Lactate (mmol·L^−1^)	3.17 ± 4.99	3.42 ± 5.30	3.25 ± 5.12	*p* = 0.406, 0.055, 19.6	***p* < 0.001, 0.80, 100.0**	*p* = 0.776, 0.02, 12.7
Glucose (mmol·L^−1^)	4.26 ± 0.53	4.25 ± 0.60	4.29 ± 0.69	*p* = 0.926, 0.005, 6.1	***p* = 0.017, 0.25, 72.4**	*p* = 0.681, 0.03, 17.2
**Hunger and Satiety Scales**						
I feel hungry (s)	5.8 ± 2.4	4.5 ± 2.1	4.5 ± 2.6	*p* = 0.295, 0.073, 25.5		
My stomach feels full	2.7 ± 1.6	4.8 ± 2.2 *	2.9 ± 2.0	***p* = 0.003, 0.309, 90.8**		
I have a desire to eat something savory	4.6 ± 2.5	4.2 ± 2.3	3.7 ± 2.5	*p* = 0.438, 0.048, 16.3		
I have a desire to eat something sweet	4.4 ± 2.4	4.5 ± 2.5	3.2 ± 2.3	*p* = 0.356, 0.062, 21.9		
I feel physically tired	5.2 ± 2.4	5.2 ± 2.3	4.5 ± 2.2	*p* = 0.606, 0.031, 12.6		
I feel sleepy/drowsy/half awake	4.7 ± 2.2	3.9 ± 2.0	4.7 ± 2.4	*p* = 0.524, 0.038, 14.4		
I feel energetic/active/lively	4.4 ± 1.6	5.1 ± 1.7	4.6 ± 1.5	p = 0.639, 0.028, 11.8		
I feel lethargic/sluggish	4.5 ± 2.1	3.1 ± 2.8	3.8 ± 2.5	*p* = 0.086, 0.142, 48.9		

Main effect of condition (NoPill, no pill; CAFF, 300 mg caffeine ingestion; or PLAC, placebo ingestion); main effect of pre vs. post warm-up for intra-aural temperature; OR main effect of sprint number for all variables associated with the repeated sprint variables measured in this study [20 and 5 m finishing time (s), heart rate, rating of perceived exertion (RPE), lactate and blood glucose (mmol·L^−1^)]; and hunger, satiety and sleep scales. *p* values, partial eta squared values (η^2^_p_) and observed power (OP) for main effects and interaction effects. **Bold** indicates significant (*p* < 0.05); underlined indicates a trend (0.1 > *p* > 0.05). * indicates > or < than either NoPill or PLAC conditions.

**Table 3:** Mean (±SD) values for subjective sleep characteristics, tiredness and alertness, profile of mood states (POMS) and caffeine withdrawal symptom (total and component scores) for the three conditions (NoPill, no pill ingestion; CAFF, 300 mg caffeine ingestion; PLAC, placebo ingestion).

Variables	NoPill	CAFF	PLAC	Significance Condition (*p*, η^2^_p_, OP)
**Sleep questions**				
Ease to sleep	−1.4 ± 2.4	−1.6 ± 2.5	−0.6 ± 2.8	*p* = 0.475, 0.045, 16.8
Get to sleep	0.9 ± 2.0	0.3 ± 2.2	0.5 ± 2.6	*p* = 0.617, 0.028, 11.1
Well Slept	−0.1 ± 2.2	−0.8 ± 2.1	−1.4 ± 2.6	*p* = 0.660, 0.025, 11.1
Waking time	−2.2 ± 2.0	−2.1 ± 2.3	−1.8 ± 1.7	*p* = 0.710, 0.021, 101
Alertness 30-min after waking	−0.5 ± 2.0	0.6 ± 2.7	−0.8 ± 1.9	*p * = 0.093, 0.138, 45.6
Tiredness (0−10 VAS)	4.9 ± 1.9	4.7 ± 2.5	4.7 ± 2.5	*p* = 0.932, 0.004, 6.0
Alertness (0−10 VAS)	5.8 ± 1.5	5.5 ± 1.5	5.8 ± 2.1	*p* = 0.731, 0.019, 9.6
Stanford sleep questionnaire	3.3 ± 0.9	2.8 ± 1.0	3.1 ± 1.0	*p* = 0.221, 0.090, 31.0
**POMS**				
Mood State−Vigour	5.9 ± 2.5	6.4 ± 3.1	6.4 ± 2.3	*p* = 0.603, 0.031, 12.7
Mood State−Anger	0.8 ± 1.1	0.5 ± 1.2	1.1 ± 2.4	*p* = 0.462, 0.038, 12.1
Mood State−Tension	0.9 ± 1.8	1.0 ± 1.7	1.1 ± 1.7	*p* = 0.802, 0.014, 8.1
Mood State−Calm	7.4 ± 2.9	7.7 ± 3.2	7.9 ± 2.6	*p* = 0.716, 0.020, 9.8
Mood State−Happiness	5.9 ± 2.5	6.6 ± 2.9	6.9 ± 3.1	*p* = 0.370, 0.060, 21.3
Mood State−Confusion	1.1 ± 2.3	1.0 ± 1.9	1.9 ± 3.0	*p* = 0.173, 0.109, 30.6
Mood State−Depression	1.7 ± 3.3	1.2 ± 2.5	1.4 ± 2.4	*p* = 0.632, 0.022, 9.7
Mood State−Fatigue	5.5 ± 4.1	5.3 ± 4.2	5.6 ± 4.1	*p* = 0.838, 0.006, 6.0
**Caffeine Withdrawal symptoms**				
Total scores	24.2 ± 8.4	23.1 ± 6.0	24.6 ± 8.3	*p* = 0.617, 0.028, 11.6
Fatigue/drowsiness	5.5 ± 3.5	4.5 ± 3.0	5.1 ± 3.6	*p* = 0.478, 0.038, 12.6
Low alertness/difficulty concentrating	7.6 ± 2.8	7.9 ± 2.4	8.8 ± 2.4	*p* = 0.144, 0.114, 39.3
Mood disturbances	2.8 ± 2.7	2.4 ± 2.4	1.9 ± 2.1	*p* = 0.401, 0.053, 17.7
Low sociability/motivation to work	5.3 ± 3.0	5.6 ± 3.2	4.9 ± 2.3	*p* = 0.634, 0.026, 11.0
Nausea/upset stomach	0.4 ± 0.9	0.4 ± 0.9	0.5 ± 0.9	*p* = 0.874, 0.008, 6.9
Flu-like feelings	1.7 ± 1.7	1.2 ± 1.0	2.0 ± 2.0	*p* = 0.257, 0.081, 28.2
Headache	0.3 ± 0.5	0.4 ± 0.9	0.2 ± 0.5	*p* = 0.507, 0.034, 11.9
Questions 21−32	3.1 ± 3.4	2.9 ± 4.1	3.0 ± 3.6	*p* = 0.948, 0.003, 5.7

**Table 4:** Comparison of the current study and other authors with the checklist for consideration for diurnal variation (DV) studies by Edwards et al. [14]. Only studies whose participants were male, consumed caffeine and used a measure of strength and/or cognitive function and were undertaken in a thermoneutral environment were included in this comparison. Abbreviations: ME = main effect; TOD = time of day; NA = not applicable; NR = not reported; WBGI = wet bulb globe temperature; Tr = core temperature; **↑ = increase**.

	Participant Considerations			Methodological and Equipment Considerations				Environment Considerations		Outcome
Author/Variable	Sample size/Sex/Age/a Priori Sample Test	Training Status/RSA Performance from Familiarization Sessions	Habitual Sleep Times/Daily Preferences for Exercise/Chronotype	RSA Assessment/Time Between Sessions/TOD of Sessions	Familiarization with Protocol/Experimental Conditions/Allocation of IDs to Sessions/Blinded or Double Blinded	Diet (Content, Timing) on Day of Testing/Caffeine Consumption from Consumption Questionnaire/Caffeine Dose and Timing	Core Temperature Site/Performance Equipment	Time of year/Sunrise and Sunset from Start to Study End	Laboratory Dry temp/Humidity/Barometric Pressure/Light Lux	ME Caffeine for Tr/ ME for Caffeine for RSA Time/ ME Caffeine for Power Output/ ME Caffeine for RPE/
Current study	n = 17/Male/20.6 ± 1.2 years/a priori = 11 or 14 for two tailed *t*-test, based on ES = 0.95 [28] and either power of 80 or 90%.	Team based recreational trained > 5 years of team participation/RSA 20 m total of 34.5 s; RSA mean 20 m time = 3.5 s.	22:54–07:36 h/Did not have one/Chronotype score = 32.4 ± 5.3 All Intermediate types	10 × 20 m sprints (30 s rest)/72 h/07:30 h	3 sessions/NoPill vs. CAFF vs. PLAC/Allocated to groups based on RAS 20 m total and mean times, then counterbalanced/double blinded	Diet analyzed by Nutritics for macro and micro content, fasting before morning session/123 mg·day^−1^/300 mg (3.7 mg·kg^−1^ body mass)	Rectal, Squirrel 1000 Series, Grant/biosynthetic runway using infrared timing gates (Brower TCi Timing system, Draper, UT, USA)	October to February (UK Autumn to Winter)/Sunrise-set range from start to end of experiment = 07:41 to 08:03 h	WBGI temperature 13–17 °C, dry temperatures of 19–21 °C/26–44% humidity/barometric pressure of 990–1022 mmHg/Ambient light of 750 lux	Tr: No change at rest (*p* = 0.709) RSA 5-m times: **No** difference (*p* = 0.552) **RSA 20-m times:** (*p* < 0.070, η^2^p = 0.18), CAFF faster than PLAC or NoPill **Fatigue Index_1_:** No difference (*p* = 0.246) **Fatigue Index_2_:** No difference (*p* = 0.912) **RSA_CHANGE_:** (*p* < 0.067, η^2^p = 0.16), > Change with CAFF than PLAC or NoPill **RPE:** CAFF values < PLAC and < NoPill; *p* = 0.024, η^2^p = 0.20)
Lopes-Silva et al. [29]	n = 13/Male/26.4 ± 4 years/a priori = NR	Physically active men/NR	NR/NR/mixed chronotypes reported	10 × 6-s cycle repeated-sprint test/time between sessions NR/08:00 h	Randomised crossover/caffeine vs. placebo in morning and afternoon/double-blind placebo-controlled	Instructed to repeat same diet 24 h prior and day of testing/NR/5 mg·kg^−1^ caffeine, 60 min before test	NR/cycle ergometer (Excalibur, Lode, Netherlands).	NR/NR	NR/NR/NR/NR	Tr: NR TW: CAFF no different compared to PLAC (*p* > 0.05) ST: CAFF no different repeated-sprint performance compared to PLAC (*p* > 0.05) PO: CAFF no different Peak power and Average power reserve compared to PLAC (*p* > 0.05) RPE: CAFF no different RPE compared to PLAC (*p* > 0.05)
Souissi et al. [28]	n = 15/Male/20 ± 1 years/a priori = 14, based on ES = 0.95.	Active men/NR	NR/NR/All intermediate types	Cognitive tests + 5-m multiple-shuttle repeated high-intensity test/six TOD sessions: 07:00, 09:00, 11:00 h	Randomised crossover/caffeine vs. placebo/allocation NR/double-blind	Isocaloric meal on day-of testing/NR/ 6 mg·kg^−1^ caffeine	NR/reaction time, attention test, 5-m multiple-shuttle run test	NR/NR	28.2–29.3 °C/45.1–46.7% /NR/NR	Tr: NR **TW: CAFF ↑ repeated high-intensity performance in the morning compared to PLAC (*p* < 0.05; exact % NR)** **ST: CAFF ↑ 5-m multiple-shuttle performance in the morning compared to PLAC (*p* < 0.05; exact % NR)** PO: NR RPE: NR

## Data Availability

The data presented in this study are available on request from the corresponding author due to privacy or ethical restrictions.
